# Universal screening or a universal risk assessment combined with risk-based screening for multidrug-resistant microorganisms upon admission: Comparing strategies

**DOI:** 10.1371/journal.pone.0289163

**Published:** 2023-07-25

**Authors:** Adriënne S. van der Schoor, Juliëtte A. Severin, Corné H. W. Klaassen, Johannes P. C. van den Akker, Marco J. Bruno, Johanna M. Hendriks, Margreet C. Vos, Anne F. Voor in ‘t holt

**Affiliations:** 1 Department of Medical Microbiology and Infectious Diseases, Erasmus MC University Medical Center, Rotterdam, The Netherlands; 2 Department of Adult Intensive Care, Erasmus MC University Medical Center, Rotterdam, The Netherlands; 3 Department of Gastroenterology and Hepatology, Erasmus MC University Medical Center, Rotterdam, The Netherlands; 4 Department of Surgery, Erasmus MC University Medical Center, Rotterdam, The Netherlands; University of Tripoli, LIBYA

## Abstract

**Objective:**

Timely identification of patients who carry multidrug-resistant microorganisms (MDRO) is needed to prevent nosocomial spread to other patients and to the hospital environment. We aimed to compare the yield of a universal screening strategy upon admission to the currently installed universal risk assessment combined with risk-based screening upon admission.

**Methods:**

This observational study was conducted within a prospective cohort study. From January 1, 2018, until September 1, 2019, patients admitted to our hospital were asked to participate. Nasal and perianal samples were taken upon admission and checked for the presence of MDRO. The results of the universal risk assessment and risk-based screening were collected retrospectively from electronic health records.

**Results:**

In total, 1017 patients with 1069 separate hospital admissions participated in the study. Universal screening identified 38 (3.6%) unknown MDRO carriers upon admission (37 individual patients), all carrying extended-spectrum beta-lactamase-producing Enterobacterales. For 946 of 1069 (88.5%) patients, both the universal risk assessment and universal screening were performed. For 19 (2.0%) admissions, ≥1 risk factor was identified. The universal risk assessment identified one (0.1%) unknown carrier, compared to 37 out of 946 carriers for the universal screening (*P*<0.001). Of the 37 carriers identified through the universal screening, 35 (94.6%) reported no risk factors.

**Conclusions:**

Our results show that in our low endemic setting, a universal screening strategy identified significantly more MDRO carriers than the currently implemented universal risk-assessment. When implementing a universal risk-assessment, risk factors should be carefully selected to be able to identify ESBL-E carriers. While the universal screening identified more MDRO carriers, further research is needed to determine the cost-effectiveness of this strategy.

## Background

Healthcare-associated infections (HAI), specifically those due to multidrug-resistant microorganisms (MDRO), are considered a worldwide threat to healthcare [1]. In hospitals, infection prevention and control (IPC) measures are implemented to prevent the spread of MDRO. However, for these measures to be effective, timely identification of patients colonized with MDRO is essential. A common IPC measure to increase timely identification is targeted screening of patients based on a universal risk assessment upon admission, followed by risk-based screening [2]. Upon admission, patients are asked several questions to determine the risk of being colonized with an MDRO and screened when they are considered at risk [2]. Another strategy is universal screening upon admission. Universal screening strategies have been performed for methicillin-resistant *Staphylococcus aureus* (MRSA), however, with conflicting results. While some studies report the method not to be cost-effective, or only effective when having a high prevalence, it has also been reported that universal screening was effective in decreasing MRSA prevalence and incidence [3–6]. Regarding carbapenemase-producing organisms, it was shown that universal screening might be a cost-effective strategy to reduce transmission [7–9]. To our knowledge, the effect of a universal screening strategy for multiple MDRO upon admission has yet to be determined.

Recently, a Dutch study showed that the nationally implemented MDRO risk assessment only identifies a small portion of all MDRO carriers, while it was associated with a high workload for healthcare workers [10]. The results of that study were confirmed by another Dutch hospital [11]. Consequently, it should be considered if other strategies are more effective. In a previous large prospective cohort study (the MOVE study), we performed universal screening for MDRO upon admission [12]. These patients were also screened with the universal MDRO risk assessment, in compliance with standard-of-care. Consequently, we are in the unique position to have patients of whom we have results of both universal screening and of the universal risk assessment combined with risk-based screening. We aimed to determine the yield of universal screening for MDRO and compare this to the yield of the currently installed universal risk assessment combined with risk-based screening, to determine the successfulness of both strategies to identify unknown MDRO carriers.

## Methods

### Study design and setting

The observational prospective cohort study (the MOVE study) was performed from January 1, 2018, until September 1, 2019, at the Erasmus MC University Medical Center (Erasmus MC) in Rotterdam, the Netherlands. The study design and setting were described previously [12]. This study was approved by the medical ethical committee of the Erasmus MC (MEC-2017-1011) and was not subject to the Medical Research Involving Human Subjects Act. Written informed consent was obtained from all participating patients. The study was registered in the Dutch National Trial Register (NL8406) [12]. Patients in the MOVE study were prospectively included. For these included patients, data on the universal risk assessment and the results of the risk-based screening was retrospectively collected from the patient’s electronic health records (EHR) between 2018 and 2022.

### Inclusion of patients for universal screening

During the study period, adult patients admitted to the participating departments at the Erasmus MC with an expected hospitalization period of ≥48 hours, and who could speak and read in Dutch, were approached for participation in the MOVE study [12]. Patients who were admitted multiple times during the study period were allowed to participate multiple times. Patients were not approached if they were admitted in the weekend/during holidays, if they were legally not able to decide about participating, or if they were in end-of-life stage [12]. After obtaining written informed consent, a nose and perianal sample were taken on the day of admission by trained members of the study team, or by the patient, with clear verbal instructions from trained members [11]. For patients admitted directly to the intensive care unit (ICU), passive informed consent was accepted (*i*.*e*., information regarding the study was provided to the patient or their family, and consent was assumed if they or their family did not explicitly object). The result of the admission screening as part of the MOVE study was not shared with the patient, nor with the treating physician, as approved by the medical ethical committee. Consequently, a positive universal screening culture did not result in isolation or otherwise change of care.

### Universal risk assessment combined with risk-based screening

The universal risk assessment combined with risk-based screening is a national mandatory assessment [2]. All patients admitted to Dutch hospitals are asked several questions upon admission to determine their risk of being colonized with MDRO (S1 File). These questions are 1) is the patient a known carrier of a MDRO, 2) has the patient recently been treated in or admitted to a healthcare institution abroad, 3) did the patient stay in a healthcare facility known with a MDRO outbreak in the past two months, and if yes, was the patient approached for screening, 4) has the patient lived in an institution for asylum seekers in the past two months, 5) does the patient live or work where pigs, veal calves or broilers are kept commercially, and 6) is the patient a partner, housemate or caregiver of someone who is MRSA positive? Additionally, in the Erasmus MC, the question “Is the patient a professional seafarer?” was added, after identifying that seafarers from the nearby harbor had higher carriage rates [13]. When patients are deemed at risk according to the assessment, screening cultures (*i*.*e*., nasal, throat, and perianal for MRSA; throat and rectal samples for other MDRO) are taken. Additionally, the patient is places in pre-emptive isolation. When a patient has had an hospitalization abroad that was more than two months ago, but has undergone surgery there or a wound is still present, screening cultures are taken, but the patient is not placed in isolation (S1 File). When the risk-based screening cultures are negative, pre-emptive isolation measures are lifted. When the screening cultures are positive, pre-emptive isolation measures are adapted to the type of MDRO. When an MDRO is identified, isolation with additional IPC measures are always initiated according to the Dutch national MDRO guideline [14]. Results of the risk assessment, results of screening cultures, and consequent implications for isolation measures, were reported in the patient’s electronic health records (EHR). These results were retrospectively collected from the EHR from patients included in the MOVE study. Consequently, from patients who were at risk according to the universal risk assessment, two types of cultures were taken: risk-based cultures and universal screening cultures as part of the MOVE study.

### Microbiological methods

Risk-based screening samples (*i*.*e*., nasal, throat, perineal, and rectal) were taken with cotton swabs. Nasal, throat, and perineal samples were screened for the presence of MRSA; rectal samples for vancomycin-resistant *Enterococcus faecium* (VRE), multidrug-resistant *Pseudomonas* spp., multidrug-resistant *Acinetobacter calcoaceticus-baumannii* complex (*A*. *baumannii*), extended-spectrum beta-lactamase (ESBL)-producing Enterobacterales (ESBL-E), and carbapenemase-producing Enterobacterales (CPE). Presence of these MDRO was determined using standard microbiological procedures (S2 File).

Universal screening samples, both nasal and perianal, were taken with flocked swabs (Copan Italia, Brescia, Italy). Nasal samples were screened for MRSA, and perianal samples were screened for VRE, highly resistant *P*. *aeruginosa*, highly resistant *A*. *baumannii*, CPE, and ESBL-E using standard microbiological procedures (S2 File). Whole genome sequencing (WGS) was performed on all isolates identified through universal screening to identify the presence of antimicrobial resistance genes (S2 File). Also, multi locus sequence types (MLST) were inferred from the WGS data. In case of discrepancy between WGS and phenotypic ESBL detection, the phenotypic test result was used, to mimic best the standard-of-care.

### Data collection and analysis

Patient data, including results from the universal risk assessment and risk-based screening, and installed isolation measures, were retrospectively collected from the EHR. Additionally, data on admission specialization was collected. Admission specializations were categorized into surgical, medical, hematological or ICU admissions [12]. Descriptive analyses were performed, and the yield of screening strategies were compared using Fisher’s exact test using IBM Statistical Package for the Social Sciences Solutions (SPSS) version 28 (IBM Corp., Armonk, New York, USA). Data was processed pseudonymized, AS and AV had access to information that could identify individual patients.

## Results

### Universal screening

**Fig 1:**
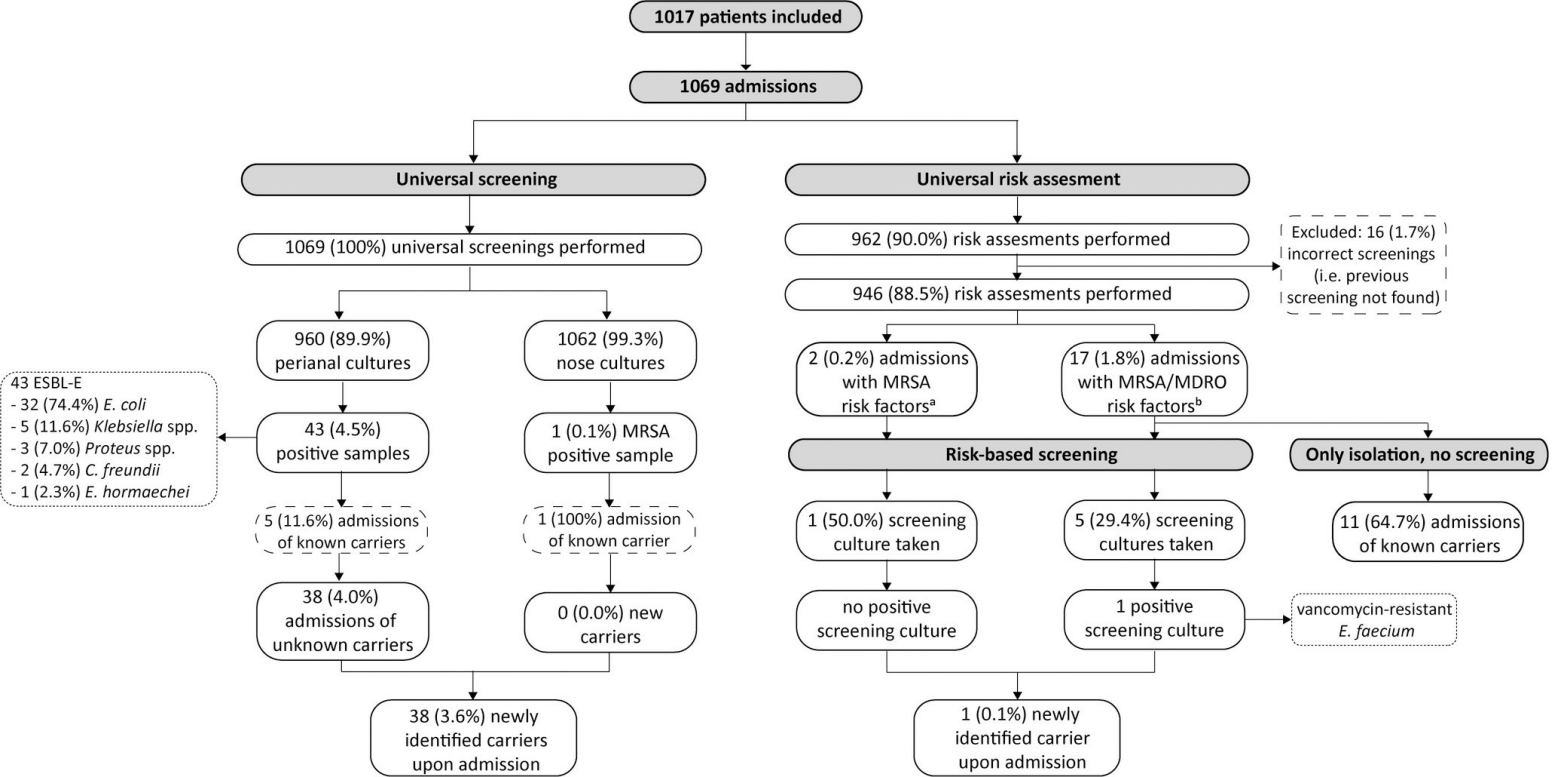
Flowchart of universal screening and universal risk assessment, using the 1069 screening results from 1017 individual patients.

In total, 1069 admission cultures were taken from 1017 patients (Fig 1). Forty-eight (4.7%) patients were admitted more than once, 44 patients were admitted twice and four patients three times. Only a nasal sample was taken for 109 (10.2%) admissions of 109 patients, and only a perianal sample was taken for seven patients (Fig 1). The median age upon admission was 61 (range 18–90). Forty-four (4.1%) cultures of 42 patients were positive for MDRO (Fig 1), 43 (4.5%) perianal cultures were positive for ESBL-E and one (0.1%) nasal culture was positive for MRSA. The majority of identified ESBL-E were *Escherichia coli* (74.4%) (Fig 1).

Six (13.6%) cultures of six patients were taken from known carriers according to their EHR, and were thus cared for in isolation. For one of 44 (2.2%) admissions, the patient was labelled as a carrier of *Citrobacter freundii* and *Enterobacter cloacae* in the EHR, both ESBL-producing, but identified as an unknown ESBL-producing *Klebsiella pneumoniae* carrier through the universal screening and thus considered as newly identified. This patient was included twice, and twice identified as a carrier of a previously unknown MDRO. Consequently, 38 out of 1069 (3.6%) admission cultures from 37 out of 1017 (3.6%) patients identified unknown carriers at the moment of hospitalization, all ESBL-E carriers. For 26 (2.4%) admissions of 26 patients, no MDRO were identified from the cultures, although patients were labelled as MDRO carrier in the EHR. Through universal screening, no carriers of VRE, CPE, highly resistant *P*. *aeruginosa* or–*A*. *baumannii* were identified. The result of WGS are presented in S3 File.

### Universal risk assessment combined with risk-based screening

The risk assessment was performed for 946 (88.5%) admissions of 900 individual patients (Fig 1). For 107 (10.0%) admissions, no risk assessment could be found in the EHR. Additionally, for 16 (1.7%) admissions, the EHR referred to a previous screening, but no previous screening was found (Fig 1). Risk factors for MDRO including the specific risk factors for MRSA were present at 19 (2.0%) admissions (Fig 1). Eleven patients were known carriers from which cultures were recently taken, and thus no screening cultures were taken, but patients were placed in isolation. For two (10.5%) admissions, risk-based screening was not performed, although risk factors were present; one patient was a roommate of or caregiver for a MRSA carrier, and one patient had been hospitalized abroad. For both patients, the risk assessment referred to a previous risk assessment, in which the risk factors were reported. The risk-based screening identified one (0.1%) new carrier. This patient was a known carrier, which was shown in cultures taken at another hospital where an outbreak occurred when the patient was hospitalized. This carrier was not identified through the universal screening. Of the 946 patients, 32 (3.4%) were a known carrier according to their EHR and were placed in isolation upon admission. Of these patients, 12 (37.5%) answered that they were a known carrier.

### Comparing screening strategies

For 946 admissions, both the universal screening and the universal risk assessment were performed. The universal screening identified 37 carriers, of which 31 new carriers, compared to the universal risk-assessment combined with risk-based screening which identified one new carrier (*P*<0.001). Thirty-five out of 37 (94.6%) carriers identified through the universal screening reported no risk factors upon admissions, two (5.4%) patients stated that they were a known carrier.

The yield of the universal screening was highest for ICU and medical patients (Table 1). These patients had the lowest percentages of performed universal risk assessments, but the highest percentages of risk factors (Table 1).

**Table 1:** Characteristics and outcome of screening strategies for the 1017 included medical, surgical, hematological, and ICU patients, with 1069 separate admissions.

	Medical (n = 203)	Surgical (n = 583)	Hematological (n = 239)	ICU (n = 44)	Total (n = 1069)
Female (%)	94 (46.3)	265 (45.5)	100 (41.8)	17 (38.6)	476 (44.5)
Age, median (range)	58 (19–90)	63 (18–89)	61 (20–81)	51 (25–85)	61 (18–90)
Labelled as MDRO carrier (%)	12 (5.9)	10 (1.7)	9 (3.8)	2 (4.5)	33 (3.1)
Universal nasal sample (%)	203 (100)	581 (99.7)	238 (99.6)	40 (90.9)	1062 (99.3)
Positive (%)	1 (0.5)	0 (-)	0 (-)	0 (-)	1 (0.1)
Universal perianal sample (%)	178 (87.7)	520 (89.2)	223 (93.3)	39 (88.6)	960 (89.8)
Positive (%)	11 (6.2)	17 (3.3)	12 (5.4)	3 (7.7)	43 (4.5)
Universal risk assessment (%)	189 (93.1)	506 (86.8)	220 (92.1)	31 (70.5)	946 (88.5)
1 risk factor (%)	6 (3.2)	7 (1.4)	4 (1.8)	1 (3.2)	18 (1.9)
≥2 risk factors (%)	1 (0.5)	0 (-)	0 (-)	0 (-)	1 (0.1)
Risk-based screening (%)	1 (0.5)	4 (0.7)	1 (0.4)	0 (-)	6 (0.6)
Positive (%)	1 (100)	0 (-)	0 (-)	0 (-)	1 (16.7)

Abbreviations: ICU, Intensive Care Unit, MDRO, multidrug-resistant microorganisms

## Discussion

Our results show that in a low endemic setting, a universal screening strategy identifies significantly more MDRO carriers than through the currently implemented universal risk assessment combined with risk-based screening.

The result that a universal screening strategy identifies more carriers in our low endemic setting is not surprising. This could be explained by the fact that more patients are microbiologically screened than through the risk-based screening. Secondly, the universal risk assessment only includes questions regarding a limited number of risk factors, and some MDRO carriers do not have any of the predefined risk factors, as shown for MRSA [15]. Since we did not identify new MRSA, CPE, highly resistant *P*. *aeruginosa* or *A*. *baumannii*, our discussion will be focused on ESBL-E. The question remains what the added benefit of identifying these MDRO (in our setting all ESBL-E) carriers would be. Several studies have studied the effect of isolation practices for known ESBL-E carriers [16, 17]. Kluytmans-van den Bergh et al. showed that transmission from index patients was higher for patients with unprotected ward stay, compared to patients who were cared for under contact precautions directly upon admission, although not significantly. This highlights the importance of timely identification and isolation [16]. Our previous study showed that most ESBL-E carriers also remain unidentified through clinical cultures throughout their hospitalization [12]. This, in combination with the high percentage of unidentified carriers upon admission, raises concern for unidentified transmissions throughout the hospital and the potential clinical implications. While we did not identify CPE, the study of Phee et al. highlighted the key role universal screening has in identifying the true prevalence of carbapenemase-producing organisms [7]. Some of the sequence types (ST) that were found among ESBL-*E*. *coli* in our study have been reported to spread in hospitals with a *bla*_NDM_ gene (ST10 in Mexico and ST167 in Denmark) [18, 19].

Our findings regarding the yield of the universal risk assessment and risk-based screening were in agreement with the study by Van Hout et al. [10] and Vainio and Bril [11]. Both our results and the results of Van Hout et al. identified that the currently installed strategy is unable to identify most carriers, and that the highest yield is through the question “are you a known MDRO carrier?”. Consequently, Van Hout et al. proposed abandoning the risk-based screening, and only installing transmission-based precautions for (previously) known carriers [10]. Vainio and Bril identified that the question about hospitalization abroad substantially contributed to the yield, and consequently they suggest a simplified risk assessment, only asking about known MDRO carrier status and recent hospitalization abroad [11]. However, according to our results, most known carriers do not report they are a known carrier. This could be deliberate, to prevent being cared for in isolation, or it could be that the patient is not aware of or does not completely understand their own MDRO status [20]. Also, due to frequent inter-hospital patient transfers, communication on the current MDRO status of a patient may be delayed. This could lead to a delay in installing isolation practices, and consequently could lead to transmission to other patients and to the hospital environment.

It is important to notice that due to transmission in the population of ESBL-E, it is difficult to implement an effective risk factor screening strategy. Not all ESBL-E carriers have (known) risk factors, which is seen in our study and in the study of Vianio and Bril, who reported that almost 80% of MDRO carriers are unexpected findings [11]. However, as shown for MRSA by Lekkerkerk et al., new risk factors can be identified [21]. Consequently, it could be worthwhile to investigate the effect of adding additional risk factors to the universal risk assessment, or to identify new risk factors for ESBL-E carriage.

### Strengths and limitations

The main strength of this study is the active sampling of patients upon admission to the hospital, regardless of risk factors or MDRO status. This study also has some limitations. The main limitation of this study is that it is a single center study in a low prevalence country. Therefore, the generalizability of our work is limited, especially to countries with a higher MDRO prevalence. A second limitation is that different cultures were taken for the universal screening compared to the risk-based screening. For example, perianal samples instead of rectal samples were taken for the universal screening. Perianal samples may be less sensitive than rectal samples for detection of MDRO, therefore, the true carriage rate upon admission may be higher than our results indicate. This could also explain why the observed carriage rate of 4.6% for ESBL-E is lower than observed in other studies in the Netherlands [16]. Additionally, it could explain why the newly identified VRE carrier was not detected in the universal screening, although it is also known that VRE colonization may be missed when only one culture is taken [22]. In general, detection of MDRO is challenging as antibiotic use of the patient or sampling error play a role, which may result in false-negative results. However, for most MDRO, we used enrichment broths to overcome this as much as possible. Another limitation is that we were not able to sample all patients admitted to the hospital. For example, patients admitted during the weekend were not approached for participation. Therefore, our results are not complete. Finally, we only included patients in the study who could speak and read Dutch.

### Future studies

Our results highlight the need for improvement of the universal risk assessment. It should be considered to add questions regarding travel history to the risk assessment, as this is a known risk factor for MDRO carriage [23–25]. Other well-known but more general risk factors, such as antibiotic usage, could be of additional value as well. To further identify risk factors and to tailor the questions, a study with MDRO carriers from multiple hospitals in countries with low prevalence of MDRO is needed. Future studies should determine if adding additional questions improves the risk assessment for patients admitted to a hospital in a low-prevalence country and if this strategy is cost-effective. Additionally, cost-effectiveness combined with the risk of transmission of identified MDRO, especially ESBL-producing *E*. *coli*, should be studied. However, as stated by Van Hout et al. [10] and by Vainio and Bril [11], the universal risk assessment is associated with a high workload for healthcare workers, and adding questions would increase this workload, which needs to be considered. A prediction system, based on data available in EHR of patients across multiple healthcare facilities, including pharmacies and general practitioners, would be a solution to overcome this in the future.

Additionally, to determine if a universal screening strategy could be an alternative strategy, the cost-effectiveness needs to be evaluated, preferably for different MDRO prevalence rates. Moreover, even though our results do not clearly show that universal screening is more effective for specific patient populations, the added benefit of universal screening for specific patient populations (*e*.*g*., ICU) should be evaluated. Our results can also be used for modelling studies to identify the best approach.

### Conclusion

Overall, our results indicate that the currently installed universal risk assessment combined with risk-based screening in a tertiary care center in the Netherlands is not successful in identifying MDRO carriers upon admission. The universal screening strategy identified significantly more new carriers. In our opinion, to improve the yield of the universal risk assessment, an updated version of the universal risk assessment would be the best approach in settings similar to ours, as the current risk factors are not identifying all ESBL-E carriers. Cost-effectiveness studies need to be performed to determine if a universal screening strategy could be a valid alternative strategy.

## Supporting information

S1 File Universal risk assessment and risk-based screening strategy. (DOCX)

S2 File Microbiological methods and whole genome sequencing. (DOCX)

S3 File Whole genome sequencing results. (DOCX)

## Abbreviations

CPE: Carbapenemase-producing Enterobacterales
EHR: Electronic health record
Erasmus MC: Erasmus MC University Medical Center
ESBL: Extended-spectrum beta-lactamase
ESBL-E: Extended-spectrum beta-lactamase-producing Enterobacterales
HAI: Healthcare-associated infections
ICU: Intensive care unit
IPC: Infection prevention and control
MDRO: Multidrug-resistant microorganisms
MIC: Minimal inhibitory concentration
MLST: multi locus sequence type
MRSA: Methicillin-resistant *Staphylococcus aureus*
ST: Sequence types
VRE: Vancomycin-resistant *Enterococcus faecium*
WGS: Whole genome sequencing
