# Supplementary material for: Universal screening or a universal risk assessment combined with risk-based screening for multidrug-resistant microorganisms upon admission: Comparing strategies
Source: PLoS One. 2023 Jul 25;18(7):e0289163. doi: 10.1371/journal.pone.0289163 (PMC10368271; doi:10.1371/journal.pone.0289163)
Supplement: S1 File — (DOCX) [file pone.0289163.s001.docx]

**Supplement 1: risk-based screening strategy questions upon admission to the hospital**


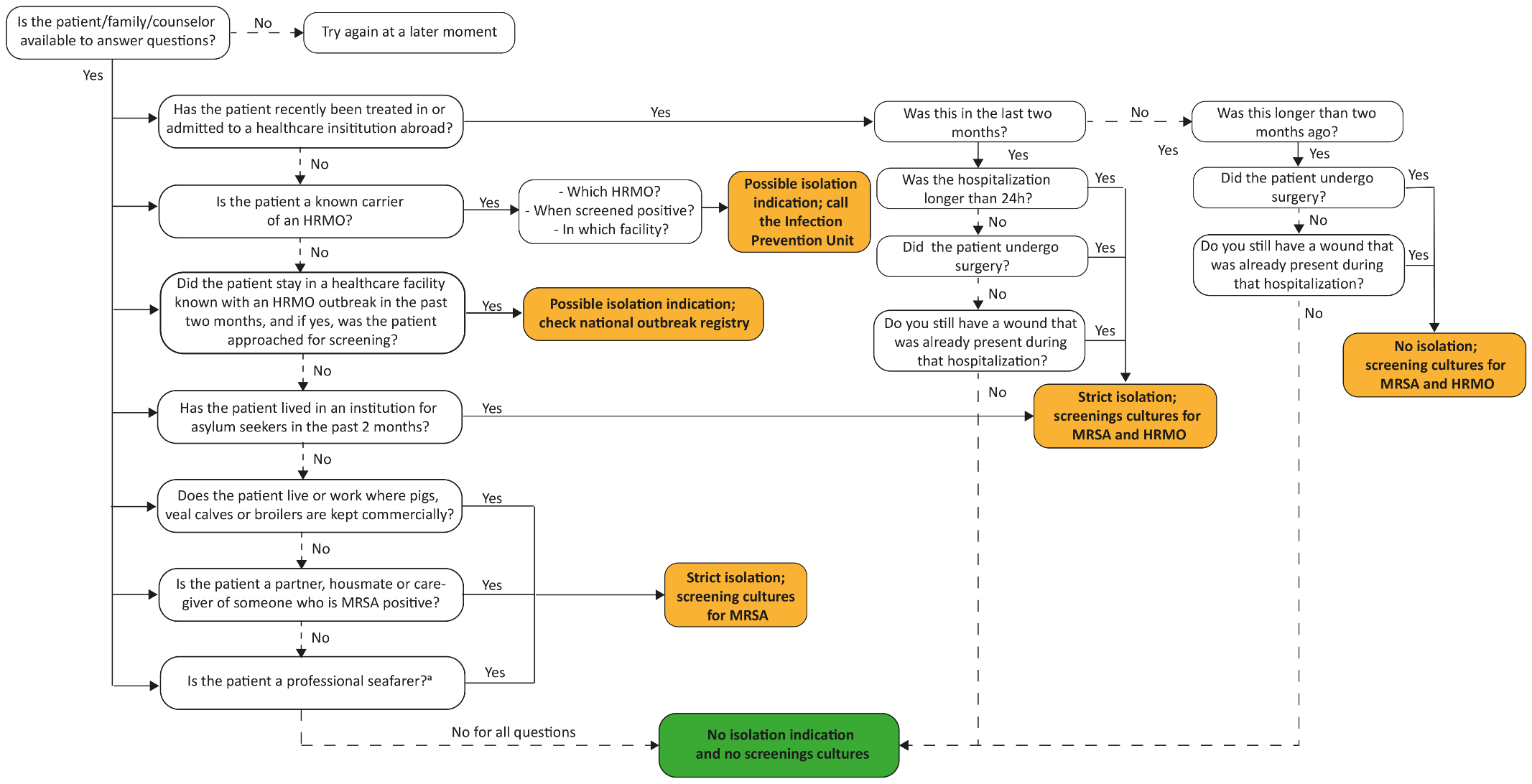


Abbreviations: HRMO highly resistant microorganisms. MRSA meticillin-resistant *Staphylococcus aureus*
^a^ Question specific for the Erasmus MC as shown by Lekkerkerk et al. (Lekkerkerk WS, van Genderen PJ, Severin JA, Peper JP, Storm EF, Vos MC. Letter to the editor: seafarers: a new risk group for meticillin-resistant *Staphylococcus aureus* (MRSA). Euro Surveill. 2013 Oct 24;18(43):20618.)
