# Supplementary material for: Universal screening or a universal risk assessment combined with risk-based screening for multidrug-resistant microorganisms upon admission: Comparing strategies
Source: PLoS One. 2023 Jul 25;18(7):e0289163. doi: 10.1371/journal.pone.0289163 (PMC10368271; doi:10.1371/journal.pone.0289163)
Supplement: S2 File — (DOCX) [file pone.0289163.s002.docx]

**Supplement 2.** Microbiological methods and whole genome sequencing

**Microbiological methods**

**Risk-based screening samples**

For methicillin-resistant *Staphylococcus aureus* (MRSA), nose, throat, and perineal samples were taken using cotton swabs (Copan Italia, Brescia, Italy). For vancomycin-resistant *Enterococcus faecium* (VRE), a rectal swab was taken. For the Gram-negative antibiotic-resistant bacteria, throat and rectal samples were taken, also with cotton swabs.

To determine the presence of MRSA, the swab was placed in a tryptic soy broth (TSB) with 6.5% NaCl and incubated for 24 h at 35°C. Subsequently, 10 µl of broth was subcultured on a BBL-CHROMagar MRSA (BD diagnostics, Sparks, USA) which was incubated for 48 h at 35°C. Plates were checked at 24 h and 48 h. Selected pink and purple colonies were identified with the Matrix-Assisted Laser Desorption/Ionization Time-Of-Flight mass spectrometry (MALDI-TOF) (Bruker Daltonics, Bremen, Germany). A suspension of 0.5 McFarland was made from *S. aureus* isolates, and used to perform a cefoxitin disk diffusion (30 µg; Oxoid, Basingstoke, UK) on a Mueller Hinton agar (BD diagnostics, Sparks, USA). A growth inhibition zone of <22mm after 18 to 24 h was considered resistant. To confirm presence of MRSA, a *mecA/mecC* PCR was performed, using established procedures.

To determine the presence of VRE, the swab was placed in an Enterococcosel broth (BD diagnostics, Sparks, USA) with 8 mg/L amoxicillin and incubated overnight at 35°C. From the broth, a *Brilliance*^TM^ VRE (Oxoid, Basingstoke, UK) was inoculated and incubated twice overnight at 35°C. Selected blue/purple colonies were identified using the MALDI-TOF and antibiotic susceptibility was determined with the VITEK®2 (bioMérieux, Marcy l’Etoile, France). For *E. faecium* colonies resistant for amoxicillin, an Etest for vancomycin, an Etest for teicoplanin, and a *vanA/vanB* PCR using established procedures, were performed to confirm the presence of VRE.

To determine the presence of extended-spectrum beta-lactamase-producing *Enterobacterales* (ESBL-E), a *Brilliance*^TM^ ESBL agar (Oxoid, Basingstoke, UK) was inoculated and incubated twice overnight at 35°C. To determine the presence of carbapenemase-producing Gram-negative bacteria, a ChromID CarbaSmart agar (bioMérieux, Marcy l’Etoile, France) was inoculated and incubated twice overnight at 35°C. All colonies were identified using the MALDI-TOF and antibiotic susceptibility was determined with the VITEK®2. ESBL production was confirmed phenotypically, using double disk diffusion test. Carbapenemase production was tested with the carbapenem inactivation method (CIM) test, presence of carbapenemase genes with a multiplex PCR for *bla*_VIM_, *bla*_IMP_, *bla*_NDM_, *bla*_KPC_ and *bla*_OXA-48-like_ (1).

To determine the presence of *bla*_OXA-48_-positive Gram-negative rods, the swab was placed in a TSB with 0.25 mg/L ertapenem and 50 mg/L vancomycin and incubated overnight at 35°C. From the broth, a PCR for *bla*_OXA-48_ was performed. In case of a positive PCR result, the broth was subcultured on a ChromID CarbaSmart agar and a MacConkey agar (Biomerieux, Marcy l’Etoile, France) and these were incubated twice overnight at 35°C. Colonies were identified using the MALDI-TOF and antibiotic susceptibility was determined with the VITEK®2.

To determine the presence of highly resistant *Acinetobacter calcoaceticus-baumannii* complex (*A. baumannii)*, the swab was placed in a TSB with 2 mg/L ceftazidime and 50 mg/L vancomycin, and incubated overnight at 35°C. From the broth, a MacConkey agar and a ChromID CarbaSmart agar were inoculated, and incubated twice overnight at 35°C. Suspected colonies were identified using the MALDI-TOF and antibiotic susceptibility was determined with the VITEK®2. To confirm the presence of highly resistant *A. baumannii*, an Etest (usually for meropenem and imipenem) or disk diffusion was performed, which was decided by the supervising clinical microbiologist.

**Universal screening samples**

Samples were taken with flocked swabs (Copan). Nasal samples were screened for MRSA, and perianal samples were screened for VRE, highly resistant *Pseudomonas aeruginosa,* highly resistant *A. baumannii*, carbapenemase-producing Enterobacterales (CPE), and ESBL-E.

Nasal samples were placed in the accompanying 2mL 2.5% NaCl TSB medium (Copan). Of the TSB medium, 800 µL was pipetted in a 6.5% NaCl TSB and incubated for 24 hours at 35°C. A *nuc* gene PCR was performed to identify the presence of *S. aureus* using established procedures. When the PCR was positive, a blood agar (BD diagnostics, Sparks, USA) was inoculated and incubated twice overnight at 35°C. Colonies were identified using the MALDI-TOF. To determine beta-lactam antibiotic resistance, a cefoxitin disk diffusion (30 µg; Oxoid, Basingstoke, UK) was performed. A growth inhibition zone of <22mm after 18 to 24 hours was considered resistant. For cefoxitin-resistant isolates, a multiplex PCR to detect *mecA* and *mecC* genes was performed using established procedures. All MRSA strains were stored in -80°C.

Perianal samples were placed in the accompanying 1 mL Amies medium ((e-Swabs (Copan)). Of the Amies medium, 250µL was pipetted in an Enterococcosel broth with 8 mg/L amoxicillin, and 250µL in a TSB with 50 mg/L vancomycin. From the amoxicillin broth, a *Brilliance*^TM^ VRE was inoculated and incubated twice overnight at 35°C to screen for VRE. From the vancomycin broth, a ChromID CarbaSmart plate was inoculated on both sides and incubated twice overnight at 35°C to screen for CPE, highly resistant *P. aeruginosa*, and highly resistant *A. baumannii*. Additionally, a *Brilliance*^TM^ ESBL agar plate was inoculated from the vancomycin broth, to screen for ESBL-E, highly resistant *P. aeruginosa*, and highly resistant *A. baumannii*. All colonies were identified to species level using the MALDI-TOF. For suspected VRE and ESBL-E, based on growth on the *Brilliance*^TM^ VRE or ESBL agar plate, respectively, antibiotic susceptibility was determined with the VITEK®2. For suspected carbapenemase-producing bacteria, based on growth on the ChromID Carba Smart, a PCR was performed to detect *bla*_VIM_, *bla*_IMP_, *bla*_NDM_, *bla*_KPC_ and *bla*_OXA-48-like_ genes using established procedures. For isolates that were negative for these carbapenemase genes, a CIM test was performed(1). All identified colonies were stored at -80°C.

**Whole genome sequencing**

WGS was performed for all identified highly resistant *P. aeruginosa,* -*A. baumannii*, CPE, ESBL-E, MRSA, and VRE isolates from universal screening samples. Total genomic DNA was extracted using the MagNA Pure 96 platform (Roche Applied Science, Mannheim, Germany). Genomic DNA was sent to Novogene (HongKong, China) where it was fragmented by shearing to a size of ~350 bp. Libraries were prepared using the NEBNext® DNA Library Prep kit (New England Biolabs, Ipswich, MA, USA) and subjected to 150 bp paired-end sequencing creating >100x coverage using Illumina technology. Fastq data were provided and *de novo* genomic assemblies were generated using CLC Genomics Workbench (Qiagen, Hilden, Germany) with default parameters (2, 3). Presence of antimicrobial resistance (AMR) genes was determined using the web-based comprehensive antimicrobial resistance database (CARD) (including perfect and strict hits)(https://card.mcmaster.ca/analyze/rgi) (4). Conventional multi locus sequence types (MLST) and core genome multi locus sequence type (cgMLST) were determined based on each species’ corresponding (cg)MLST scheme (https://cgmlst.org/ncs) available in SeqSphere+ software (Ridom, Munster, Germany). Isolates were identified to the species level by analysing their de-novo assemblies using the Tyge Strain Genome Server (TYGS - <https://tygs.dsmz.de/>) (5).

**References**

1. van der Zwaluw K, de Haan A, Pluister GN, Bootsma HJ, de Neeling AJ, Schouls LM. The carbapenem inactivation method (CIM), a simple and low-cost alternative for the Carba NP test to assess phenotypic carbapenemase activity in gram-negative rods. PLoS One. 2015;10(3):e0123690.

2. Wick RR, Judd LM, Gorrie CL, Holt KE. Unicycler: Resolving bacterial genome assemblies from short and long sequencing reads. PLoS Comput Biol. 2017;13(6):e1005595.

3. Seemann T. Prokka: rapid prokaryotic genome annotation. Bioinformatics. 2014;30(14):2068-9.

4. Jia B, Raphenya AR, Alcock B, Waglechner N, Guo P, Tsang KK, et al. CARD 2017: expansion and model-centric curation of the comprehensive antibiotic resistance database. Nucleic Acids Res. 2017;45(D1):D566-D73.

5. Meier-Kolthoff JP, Göker M. TYGS is an automated high-throughput platform for state-of-the-art genome-based taxonomy. Nat Commun. 2019;10(1):2182.
