# Supplementary material for: Universal screening or a universal risk assessment combined with risk-based screening for multidrug-resistant microorganisms upon admission: Comparing strategies
Source: PLoS One. 2023 Jul 25;18(7):e0289163. doi: 10.1371/journal.pone.0289163 (PMC10368271; doi:10.1371/journal.pone.0289163)
Supplement: S3 File — (DOCX) [file pone.0289163.s003.docx]

**Supplemental file 3. Whole genome sequencing results**

WGS showed that the majority of *Escherichia coli* (97%) and *Klebsiella* spp. (100%) isolates were CTX-M positive (Supplementary file 3). Of the 32 ESBL-*E. coli*, 11 (34.4%) belonged to sequence type (ST) 131. One *Citrobacter freundii* carried an *mcr*-9 gene (with a colistin minimal inhibitory concentration (MIC) of 0.5 µg/mL as measured by Vitek2). While the *Proteus* spp. phenotypically showed ESBL activity, no beta-lactamase resistance genes were identified (Supplementary file 3).

Prevalence of antimicrobial resistance genes among the different species as determined using the CARD web-interface (https://card.mcmaster.ca/). The analysis (restricted to perfect and strict hits only) was focused on different types of beta-lactamases and aminoglycoside modifying enzymes.

*sequence types involved: E.coli ST131 (n=11), ST10/ST69 (n=4), ST405/ST744 (n=2), ST38/ST58/ST75/ST88/ST120/ST167/ST224/ST636/ST13164 (n=1); *K. pneumoniae* ST152/ST307/ST464/ST561 (n=1); S. aureus ST6 (n=1)
